# Study protocol for patient response to spinal manipulation – a prospective observational clinical trial on physiological and patient-centered outcomes in patients with chronic low back pain

**DOI:** 10.1186/1472-6882-14-292

**Published:** 2014-08-08

**Authors:** Ting Xia, David G Wilder, Maruti R Gudavalli, James W DeVocht, Robert D Vining, Katherine A Pohlman, Gregory N Kawchuk, Cynthia R Long, Christine M Goertz

**Affiliations:** Palmer Center for Chiropractic Research, Palmer College of Chiropractic, 741 Brady Street, Davenport, Lowa, IA 52803 USA; Department of Biomedical Engineering, University of Iowa, Lowa City, Lowa USA; Department of Pediatrics, University of Alberta, Edmonton, AB Canada; Department of Physical Therapy, University of Alberta, Edmonton, AB Canada

**Keywords:** Low back pain, Spinal manipulation, Patient-centered outcomes, Spinal stiffness, Flexion relaxation, Spine segmental load

## Abstract

**Background:**

Low back pain (LBP) is a major health issue due to its high prevalence rate and socioeconomic cost. While spinal manipulation (SM) is recommended for LBP treatment by recently published clinical guidelines, the underlying therapeutic mechanisms remain unclear. Spinal stiffness is routinely examined and used in clinical decisions for SM delivery. It has also been explored as a predictor for clinical improvement. Flexion-relaxation phenomenon has been demonstrated to distinguish between LBP and healthy populations. The primary objective of the current study is to collect preliminary estimates of variability and effect size for the associations of these two physiological measures with patient-centered outcomes in chronic LBP patients. Additionally biomechanical characteristics of SM delivery are collected with the intention to explore the potential dose–response relationship between SM and LBP improvement.

**Methods/Design:**

This is a prospective, observational study applying side-lying, high velocity, low amplitude SM as treatment for patients with LBP over a course of 6 weeks. Approximately 80 participants will be enrolled if they present with chronic LBP of 1, 2 or 3 in Quebec Task Force Classification for spinal disorders, a Roland-Morris Disability Questionnaire (RMDQ) score ≥ 6, and persistent LBP ≥ 2 with a maximum ≥ 4 using numerical rating scale. Patient-centered outcomes include LBP using visual analog scale, RMDQ, and PROMIS-29. Lumbar spine stiffness is assessed using palpation, a hand-held instrumented device, and an automated device. Flexion-relaxation is assessed using surface electromyography at the third level of the lumbar spine. Biomechanical characteristics of SM are assessed using a self-reported, itemized description system, as well as advanced kinetic measures that will be applied to estimate forces and moments at the lumbar segment level targeted by SM.

**Discussion:**

Beside alterations in material properties of the passive components of the spine, increased neuromuscular activity may also contribute to a stiffened spine. Examining changes in both spinal stiffness and flexion-relaxation along the course of the treatment provides an opportunity to understand if the therapeutic effect of SM is associated with its action on active and/or passive components of the spine.

**Trial registration:**

NCT01670292 on clinicaltrials.gov.

## Background

Low back pain (LBP) is a major public health problem due to its high prevalence and staggering socioeconomic impact [1–5]. The pathophysiology of chronic LBP in particular is not well understood, with approximately 90% of cases categorized as either idiopathic or non-specific [6, 7]. Conservative approaches for LBP treatment include medication, exercise, and manual therapy [8]. Spinal manipulation (SM), a form of manual therapy delivered by physical therapists, osteopathic physicians and most commonly by doctors of chiropractic (DCs), has been recommended by recently published clinical practice guidelines as an effective treatment option for LBP [8, 9]. Systematic reviews of clinical trials demonstrate that SM has therapeutic effects comparable to other non-invasive treatment methods such as physical therapy and core muscle exercise [10–13].

In the U.S., at least 8% of the population seeks care from DCs annually, representing approximately 190 million patient visits [14]. Among patients reporting back or neck pain, 20% seek chiropractic care [15]. Additionally, patients are highly satisfied with chiropractic care [16, 17]. In spite of its relatively widespread use by the public, the underlying therapeutic mechanisms of SM are largely unknown. Thus, it is important to investigate physiological measures that may serve both as markers of LBP severity and clinical response to SM. Two such measures are spinal stiffness and the flexion-relaxation phenomenon.

Manual therapy practitioners routinely assess spinal stiffness (e.g. the perceived spinal resistance to manually applied force) as one component of the clinical evaluation [18]. Combined with information from the overall clinical picture, DCs may elect to employ SM targeted toward regions of higher resistance with the intention to improve mobility, which in turn is believed to contribute to improved clinical outcomes [19, 20].

Studies show increased spinal stiffness using instrumented stiffness measurements in patients with LBP compared to those who are asymptomatic [21, 22]. One recent study by Fritz et al. [23] suggests that spinal stiffness assessed using an automated device may be a valuable predictor of clinical outcome for patients with LBP [23]. In particular, the authors found an immediate decrease in spinal stiffness following the first treatment and lower baseline spinal stiffness were associated with better Oswestry disability index scores after 1 week of SM treatment. However, there are conflicting reports regarding whether or not SM can reduce spinal stiffness [23–28].

The flexion-relaxation phenomenon (FRP) provides information regarding the nature of neuromuscular functioning in patients with LBP [29]. It is characterized by relaxation of lumbar paraspinal muscles during full torso flexion while standing. The muscular relaxation is likely associated with the principle of energy conservation as passive tissues are thought to support and stabilize the spine at the end range position without the need for active muscle contraction [30]. The phenomenon can readily be seen by visual inspection of a plot of electromyographic (EMG) data taken from the lumbar paraspinal musculature while a participant stands erect, bends forward as far as he/she can, maintains the fully flexed position briefly, and then returns to the upright position. For asymptomatic individuals, the plot typically shows very little muscular activity while the participant stands erect, an increase in activity while bending forward due to eccentric paraspinal muscle contraction, very little activity while fully flexed, and a greater activity increase while arising due to concentric muscular contraction. Several ways of quantifying the FRP have been reported in the literature, although no one method has emerged as universally accepted [31, 32]. In general, comparisons have been made between 1) the highest muscle activity recorded while the participant is bending forward vs. the mean value while fully flexed, and 2) the highest muscle activity recorded while extending to an upright position vs. the mean value while fully flexed.

The phenomenon is most clearly seen in patients without LBP [33]. The absence of or a reduced FRP, indicating paraspinal muscle activation instead of relaxation during full torso flexion, is thought to represent neuromuscular system dysfunction, and is typical in patients with LBP [29]. Based on a review of the literature, there are indications that FRP may be a valuable objective clinical tool to aid in the diagnosis and treatment of patients with LBP [34].

Given the above, the primary objective (i.e. Specific Aim 1) of the current study is to collect preliminary estimates of variability and effect size for the associations of these two physiological measures with patient-centered outcomes in patients with chronic LBP. Study findings will aid us in planning properly-powered clinical trials that examine if baseline lumbar spine stiffness and flexion-relaxation 1) are associated with baseline patient-centered outcomes; 2) predict improvement in patient-centered outcomes after treatment with SM; and 3) improve along the course of treatment.

Secondary objectives (i.e. Specific Aim 2) will explore the therapeutic mechanism of SM from a dose–response perspective. We will obtain preliminary estimates of variability and effect size to determine if differences in spinal manipulation delivery, as estimated by thrust contact force and spinal segment load, are related to patient-centered outcomes. Clinicians use a variety of SM procedures but most commonly contact the patient with their hands while delivering quick dynamic loads at specific locations of the spine with a duration range of 100–500 milliseconds [35–39]. These quickly applied dynamic forces are known as high velocity, low amplitude SM (HVLA-SM). Because considerable variation exists in HVLA-SM in terms of rate of loading, pre-load, peak force, and duration of loading, quantification and standardization of delivery may be important for maximizing therapeutic effects [38, 40–42]. In the current study, DC self-reported descriptions of HVLA-SM and lumbar spine segmental load estimation methods have been developed and implemented to explore this area. Additionally, dynamic spinal stiffness during SM is explored by measuring apparent mass and driving-point impedance [43, 44].

## Methods/Design

### Study population

Ethics approval was obtained from the Palmer College of Chiropractic Institution Review Board (IRB) with the Assurance Number - X2011X141. Participants are eligible if they are between 21–65 years old, meeting the classification of 1, 2, or 3 in Quebec Task Force (QTF) classification for Spinal Disorders [45, 46], present with LBP for more than 12 weeks, have a Roland Morris Disability Questionnaire (RMDQ) ≥ 6 at phone screen, and a self-reported average pain within the past 24 hours ≥ 2 at phone screen and baseline visits 1 and 2 (BL1 and BL2), and ≥ 4 at phone screen or BL1 on an 11 point numerical rating scale (NRS). A more detailed description of the inclusion/exclusion criteria is summarized in Table 1. Potential participants are recruited using a multimedia approach of newspaper ads and direct mail promotions that have been used in our previous LBP studies [47–49]. Additionally press releases, media interviews, a local newspaper website, community flyers, roadside signs, Craigslist, and other online classified ads are used. All advertisement methods involved in the recruitment process have been approved by the Palmer College of Chiropractic IRB.

**Table 1:** **Study inclusion and exclusion criteria**

Inclusion criteria	Rationale	Source
Age ≥ 21 and ≤ 65	Chronic LBP not as common under age 21. Older adults not as likely to tolerate biomechanical tests	PS, BL1
Chronic low back pain matching QTF Classifications 1, 2, and 3	Low back pain, uncomplicated by known nerve root compression, neurological signs or prior surgery	CR
RMDQ ≥ 6	Disability high enough to prevent floor effect	PS
NRS Pain (Average within past 24 hours) ≥ 2 at PS and BL1 and BL2, and ≥ 4 at PS or BL1	Pain high enough to prevent floor effect	PS, BL1, BL2
**Exclusion criteria**		
Additional diagnostic tests or urgent/emergent procedures needed beyond study exam procedures	Advanced diagnostic tests or other necessary evaluation(s) are outside study scope	CR
BMI ≥ 40	Unable to adequately perform manipulation procedures per study protocol	BL1
BDI II score ≥ 29	May interfere with protocol compliance and data collection	BL1
Compliance concerns	May compromise ability to comply with study protocols	CR
Co-morbidity requiring coincident clinical management	May interfere with study requirements, pose significant scheduling burden, or pose a safety risk	CR
Inability to read or verbally comprehend English	Proxy unavailable	BL1
Inflammatory or destructive tissue changes to the spine	Potential intolerance to biomechanical testing or treatment protocols	PS, CR
Joint replacement history	Potential intolerance to biomechanical testing or treatment protocols	PS, CR
Moving from area within 8 weeks	May interfere with ability to comply with study protocol	PS, BL1, BL2
Neuromuscular disease	Interference with biomechanical measurements	PS
No indication for SM at L1 – L5 or sacroiliac joint (s)	Spinal Manipulation is only treatment available	CR
Open or pending litigation for LBP or seeking/receiving disability compensation	May interfere with study compliance or data collection	PS, BL1
Pacemaker/Defibrillator	Safety due to potential electromagnetic fields produced by biomechanical testing equipment	PS, BL1
Peripheral arterial disease	Potential intolerance to biomechanical tests, potential need for referral, and interference with pain and disability measures	PS, CR
Pregnancy	Safety for biomechanical testing and may interfere with data collection	PS, BL1
QTF classification 4-11	Conditions sufficiently complicated to cause intolerance to biomechanical testing procedures or data collection	CR
Received SM within past 4 weeks	May interfere with data interpretation	BL1
Safety	Precaution for condition(s) posing a safety risk or intolerance for treatment or biomechanical tests (i.e., excessive bruising/bleeding and adhesive sensitivity)	CR
Suspicion of drug or alcohol abuse	May interfere with data collection, ability to comply with study protocol, and require referral	CR
Uncontrolled hypertension	May interfere with study protocols and require referral	CR

BDI-II: Beck Depression Inventory; BL1: Baseline Visit 1; BL2: Baseline Visit 2; BMI: body mass index; CR: Case Review; L1 – L5: lumbar segment level 1 to 5. LBP: low back pain; NRS: numerical rating scale; PS: Phone Screen; QTF- Quebec Task Force; SM: Spinal Manipulation.

### Study design

The current study is a prospective observational trial to evaluate the delivery of HVLA-SM using patient-centered outcomes and physiological measures over a course of 6 weeks. A total of 80 participants are projected to be recruited over a period of 20 months (September 2012 through May 2014, roughly 4 participants per month). Primary outcomes include patient-centered outcomes of back pain and function and physiological measures. Physiological measures consist of posterior-anterior stiffness of the lumbar spine using three assessment methods and flexion-relaxation. The assessment for physiological measures is implemented in the following sequence: lumbar spine stiffness measurements using hand palpation, a hand-held instrumented stiffness device, an automated stiffness device, and flexion-relaxation. The sequence was chosen to improve visit efficiency and reduce participant burden. Secondary outcomes include the descriptive record of SM delivered in each treatment visit (TV) and lumbar spine segmental load during SM delivery. Demographic and clinical characteristics of the participants are collected during BL1.

#### Patient visit protocol

Participant screening begins with an initial eligibility determination via phone screen. Interested and eligible participants are scheduled for an in-person BL1 consisting of an informed consent process and comprehensive clinical examination. Following the BL1 a panel renders eligibility decisions for criteria requiring clinical decision-making in a process called case review, as described in more details below. Final eligibility determination and enrollment for participants still eligible occurs during BL2. The treatment phase consists of 2 TVs per week over an approximate 6 week period. The BL2 (pre enrollment) and TV1 (post enrollment) occur on the same day. Patient-centered outcomes are collected at BL1, TV6, and TV13. The physiological assessments are performed immediately before and after SM delivery during TV1, TV5, and TV12 (6 sets of assessments in total). During these same TVs, full kinetic measures during SM delivery are captured for lumbar spine segmental load analysis. SM thrust profiles (i.e. simple kinetics including contact force and displacement and acceleration at DC’s thrusting hand) are assessed separately at TV3 and TV9. An exit interview occurs at the 13th TV following the completion of treatment and all study related activities. The detailed visit-by-visit activities are summarized in Table 2.

**Table 2:** **Summary of clinical activities in the visit-by-visit basis**

	Visit	Activities
Screening	Phone screen	Computer‒assisted telephone interview
	Baseline visit 1	Informed consent document
		Study flow chart
		Study procedure video
		HIPAA
		Patient-centered outcomes
		Past medical history
		Medication checklist
		Demographics & Vital signs
		Examination
		X-ray & Urinalysis, if indicated
		Case review
	Baseline visit 2	Report of findings, enroll
Treatment	Treatment visit 1	Pre-treatment Pain in VAS
		Physiological assessments
		Treatment-full kinetics
		Physiological assessments
		Post-treatment pain in VAS
	Treatment visit 2	Pre-treatment pain in VAS
		Treatment
		Post-treatment pain in VAS
	Treatment visit 3	Pre-treatment pain in VAS
		Treatment-simple kinetics
		Post-treatment pain in VAS
	Treatment visit 4	Same as treatment visit 2
	Treatment visit 5	Same as treatment visit 1
	Treatment visit 6	Patient-centered outcomes
		Pre-treatment pain in VAS
		Treatment
		Post-treatment pain in VAS
	Treatment visits 7-8	Same as treatment visit 2
	Treatment visit 9	Same as treatment visit 3
	Treatment visits 10-11	Same as treatment visit 2
	Treatment visit 12	Same as treatment visit 1
Exit interview	Treatment visit 13 (Final)	Patient-centered outcomes
		Satisfaction questionnaire
		Exit interview

HIPAA: Health Insurance Portability and Accountability Act; VAS: visual analog scale.

#### Eligibility determination

Interested individuals contact our center by phone or with a return pre-stamped postcard. Study personnel administer a short computer-assisted telephone interview to screen for provisional eligibility and, if eligible and still interested, schedule a BL1 visit. During a BL1visit, a study coordinator reviews the informed consent document, study flow chart, and specific visit activities with the participant. A short video describing study procedures is also viewed. The participant has opportunity to read the informed consent document and ask questions. Those who wish to further participate sign the written informed consent document. Vital signs, height, and weight are then measured by a study coordinator and the participant completes several research forms including patient-centered outcomes, demographics, Beck Depression Inventory, a substance assessment questionnaire, medication use, and a health history. Study coordinators review forms for incomplete data and answer computer-based questions programmed with eligibility criteria.

A DC reviews health-related research documents, noting where further information is needed to determine eligibility. The DC then conducts a focused low back diagnostic examination by first obtaining a focused LBP history. Lumbar spine X-rays and/or urinalysis may be obtained to assist in diagnosis and provide information to render a safety determination. If additional laboratory procedures or diagnostic tests are required to evaluate the participant’s LBP or health status, he or she is excluded and referred to an appropriate healthcare provider. With participant consent, other health records may be requested and reviewed to determine eligibility. Following the BL1, participants are scheduled for the BL2.

The clinician who performed the BL1 examination presents findings to a Case Review panel consisting of research clinicians, study coordinators, and at least one investigator. The Case Review panel renders eligibility determination for criteria requiring subjective and clinical decision-making. Case Review provides a formally structured eligibility determination process including at least 1 person from each of 3 roles: the consenting coordinator, research clinician, and investigator to: 1) facilitate consistent interpretation of eligibility criteria; 2) ensure participant safety; and 3) mitigate selection bias. This research team has used a similar case review process to determine eligibility in previous clinical trials [47, 49]. Following eligibility determination, a web module programmed with explicit exclusion criteria is completed for each participant. Eligible participants proceed to the BL2. Participants no longer eligible receive a phone call from the examining clinician who informs them of the determination, a summary of the exam findings and appropriate clinical recommendations.

### Spinal manipulation intervention

A standardized side lying, HVLA-SM is the treatment procedure utilized. Participants lie in a lateral recumbent position with the lower leg straightened and the superior or free leg flexed at both the hip and knee and adducted across midline. The DC, standing and facing the participant, stabilizes the superior shoulder or upper arm with one hand (i.e. non-thrusting hand) as the participant’s forearms rest across the chest or abdomen. The participant’s free leg is stabilized by the DC’s thigh or lower leg (i.e. contacting leg, the same side as the thrusting hand). The manipulative load is delivered by a quick, and short controlled movement of the DC’s shoulder, arm, and hand in combination with a slight body drop. The areas where the thrusting hand contacts the participant include lumbo-pelvic tissues over or adjacent to vertebral mammillary and spinous processes, between and slightly lateral to the posterior superior iliac spine of the ilium, ischial tuberosity, and the sacrum. The manipulative load is also transmitted to participants through the clinician’s stabilizing thigh/leg, resulting in a twisting force directed to the pelvis. In the current study the thrust is only delivered using the palmar aspect of the hand. Other side lying HVLA-SM procedures utilizing other manual contacts, such as pulling on a lumbar spinous process with fingers, are not utilized. The HVLA-SM is delivered by a team of trained and experienced clinicians who have at least 15 years of clinical experience using HVLA-SM for treating patients with LBP. To enable the assessment of segmental load at the lumbosacral region during the TV1, TV5, and TV12, the patient’s upper body is restrained by an external strap system while the clinician places the non-thrusting hand on a rest located near the participant’s free shoulder [50, 51].

### Primary outcome measures

#### Patient-centered outcomes

The primary patient-centered outcomes include the Visual Analog Scale (VAS) for LBP, the 24-item RMDQ, and the Patient Reported Outcomes Measurement Information System (PROMIS)-29 questionnaire. The RMDQ has been validated in previous studies in patients with LBP and is more responsive to change over time than most other functional status measures [52–54]. The VAS is used to evaluate the worst, least, and average LBP over the past 24 hours using a 100 millimeter horizontal scale (0 = no pain; 100 = worst imaginable pain). Current pain is also monitored with the VAS at the beginning and end of each TV. The VAS has excellent metric properties, is easy to administer and score, and has received much use in LBP research [11]. The comprehensive LBP status is evaluated using the PROMIS-29 questionnaire, which is a collection of questions measuring physical function, anxiety, depression, fatigue, sleep disturbance, satisfaction with social role, pain interference and pain intensity. It aims to provide clinicians and researchers access to efficient, precise, valid, and responsive adult- and child-reported measures of health and well-being [55]. Biomechanical assessors and treating clinicians are blinded to patient-centered outcomes data.

#### Posterior-anterior stiffness of the lumbar spine

Lumbar spine stiffness is assessed sequentially with three approaches using hand palpation, a hand-held instrumented device, and an automated device, respectively. The hand-held instrument approach is the primary measure as it has been validated by our study team and demonstrated good reliability (0.79) [56, 57]. The same set-up allows us to implement hand palpation in a manner that mimics spinal stiffness assessments in clinical practice and will allow us to explore the association between stiffness as measured by palpation versus the hand-held instrument. Because the hand palpation approach was thought to be less intrusive, it is tested before the hand-held approach in the trial. As we finalized the protocol for study launch, a study using an automated stiffness assessment method with high within- and between-day reliabilities (0.99 and 0.98) was published and suggests that spinal stiffness may have important clinical implications [23]. We contacted the authors who loaned us the equipment for use as an added stiffness measure. However, due to the study timeline, and technical and training requirements, the automated stiffness procedure was not implemented until the clinical trial enrolled 20 participants.

**Figure 1:**
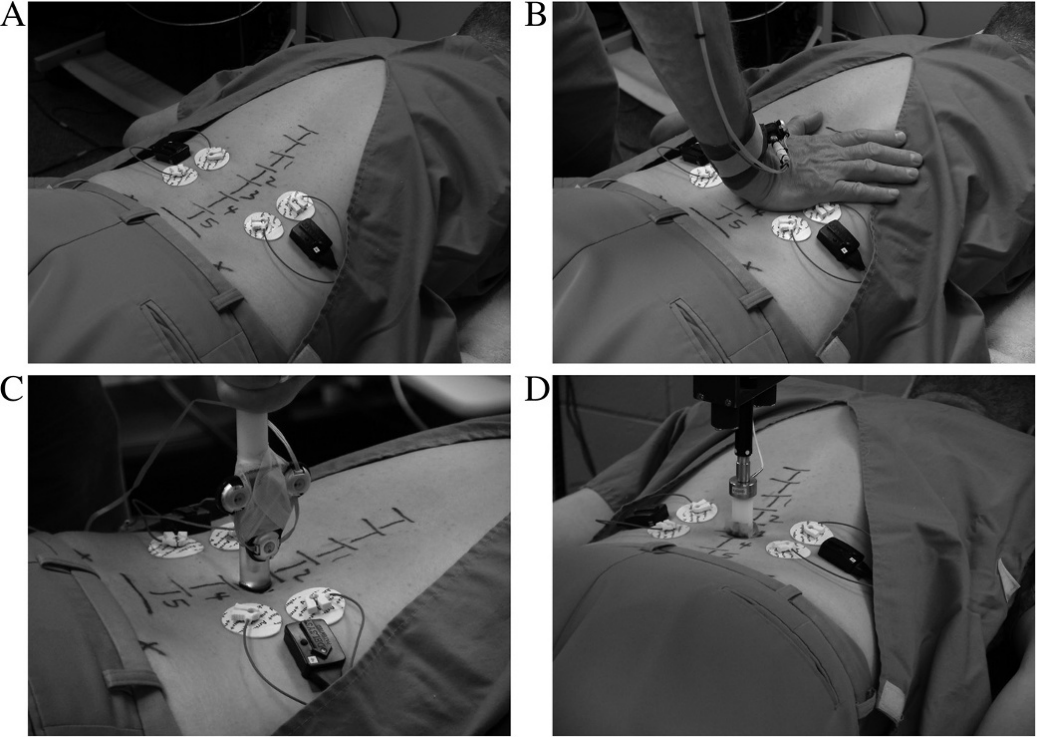
**Illustration of spinal stiffness assessment tests.** Skin marks over bony landmarks of the T11-L5 spinous processes and posterior superior iliac spine **(A)**, palpatory stiffness assessment **(B)**, stiffness assessment with a hand-held device **(C)**; and stiffness assessment with an automated device **(D)**.

During stiffness assessments, participants lie prone while study clinicians mark the skin with a sterile surgical marking pen at the following locations: posterior superior iliac spines, spinous processes the seventh cervical vertebra (C7), the eleventh thoracic vertebra (T11) to the fifth lumbar vertebra (L5), interspinous spaces from T10 to the first level of sacrum (S1), and the most concave lumbar segment while lying prone (Figure 1A). During the hand palpation assessment, the participants lie face down on a custom-made chiropractic treatment table with force plates (Model 4060-NC, Bertec Inc., Columbus, OH) embedded under the cushions that support the thoracic and pelvic regions and acquire the palpatory force transmitted through the participant. The clinician applies a gentle anteriorly directed force consistent with the level typically used during examination to the vertebral levels L1 to L5. An infrared smart marker (Optotrak 3020/Certus hybrid system, Northern Digital Inc., Waterloo, Ontario, Canada) is placed on the clinician’s palpatory hand to simultaneously acquire displacement. The sampling rate for the Optotrak system is set at 100 Hz while the sampling rate for the force plates is set at 1000 Hz. No specific instruction is given to participants in terms of their breathing during the test. Figure 1B demonstrates the setup for the palpatory stiffness measurement.

The hand-held stiffness device consists of a force transducer (Model # LC201-50, Omegadyne Inc., Sunbury, OH) and infrared smart markers for accurate force and displacement measurement. The assessment is performed with the participant lying prone on the treatment table. The examiner assesses tolerance of participants by pressing gradually up to a maximum of 80 N with the stiffness device in an anterior direction over the pre-marked spinous process of each lumbar vertebra from L1-L5. Participants inform the examiner if the pressure causes discomfort. When the maximum pressure of 80 N is reached an audible tone sounds as feedback to the examiner to stop compressing and withdraw the device. If pressure from the stiffness device causes discomfort, the test is reapplied more slowly and the participant indicates the level of pressure causing discomfort. If the tolerance test does not cause discomfort with 80 N of pressure at any vertebral level, the subsequent stiffness tests are conducted with a maximum pressure of 80 N. If the participant indicates discomfort during the tolerance test, the subsequent stiffness test is conducted at all vertebral levels using a maximum force value 10 N lower than the lowest value causing discomfort at any vertebral level during the tolerance test. If tolerance to the hand-held stiffness device is less than 60 N at any vertebral level, the hand-held stiffness test, as well as the automated stiffness test, is not performed. Both force and displacement are recorded using a Motion Monitor data acquisition workstation (Innovative Sport Training, Chicago, IL) at a sampling rate of 1000 Hz. The stiffness measures are performed by pressing anteriorly over each lumbar spinous process for 5 cycles at a rate of approximately 1 repetition per second. The participants are instructed to make a deep breath in, a deep breath out, and then inhale half way and hold their breath during which the test is performed. The average stiffness obtained over the last 4 cycles is used for analysis. Figure 1C demonstrates the setup for the hand-held stiffness measurement.

The automated stiffness test consists of 1) a height-adjustable treatment table equipped with safety switches to allow either the participant or examiner to immediately lower the table thereby withdrawing from the automated stiffness device; 2) an automated stiffness device that extends and applies a designated force; and 3) a computer system to program device movement based on displacement/force data sent from the device in real-time using a custom-written program in LabVIEW with a sampling rate at 200Hz (Version 8, National Instruments Corp., Austin, TX) [23, 58, 59]. The automated stiffness device is positioned over the spinous process nearest the most concave point of the lumbar curve while in the prone position, typically corresponding with the L3 spinous process. When the lumbar spine is not concave in shape (e.g. flat or convex lumbar spine) or the L2 or L4 level appears to be at similar height as L3, the automated stiffness device is placed over the L3 spinous process. Participants are instructed to lay prone, inhale then exhale normally, and hold their breath at the end of exhalation for approximately 10 seconds during which the automated stiffness test is performed. During the test, the actuator on the automated device advances at a rate of 2.5 mm per second. After reaching a preload force of 5 N on the target spinous process, the actuator holds the preload for one second before advancing further and applying a maximum load of 60 N. The maximum load is maintained for one second before the actuator retracts. To decrease participant’s burden in holding the breath, the initial position of the actuator is placed as close as possible to the target spinous process as long as the participants can breathe normally without pushing against the actuator. The procedure is conducted three times during each assessment with a testing trial performed prior to data collection to orient the participant to the procedure and to determine safety. The average stiffness of the three trials is used for analysis. Figure 1D demonstrates the setup for the automated stiffness measurement.

*Data reduction*: For all three stiffness measurement tests, force and displacement data are acquired simultaneously. Data acquired from the hand palpation and hand-held stiffness tests are exported into a custom-written MATLAB program (MathWorks Inc., Natick, MA) and the graphs of displacement against force are plotted. The user identifies the points corresponding to 10 N and 60 N and the software computes the slope of the curve within the region using linear regression. For the automated stiffness test, the LabVIEW program is used to compute stiffness using the same algorithm as that of the MATLAB program. It is worth noting that spinal stiffness obtained *in vivo* in the current study is different from the inter-segmental stiffness typically obtained in specimen studies. To distinguish between them, the term global stiffness was suggested to describe the stiffness measurement over a region of the spine containing several segments [60]. Consultation with DCs performing treatments in this study revealed that relative perceived stiffness as opposed to the absolute stiffness of a given spinal segment provides the most useful clinical information. Therefore, to be consistent with the clinical interpretation utilized by study DCs the global stiffness variation (GSV) is calculated as:


where gs_i_ is the global stiffness measured at the lumbar spine and *i* = 1,2,…,5.

To account for the between-subject variation in the global stiffness measurement (e.g. a participant may have an overall stiffer or more flexible lumbar spine), the normalized *GSV* (*nGSV*) is calculated as:


Both *GSV* and *nGSV* are obtained from the palpatory and hand-held stiffness tests and will be used in data analysis.

#### Flexion-relaxation phenomenon

Surface EMG measurements of lumbar paraspinal muscle activity for FRP analysis in this study were initially taken with a system using Ag/AgCl electrodes built into a plastic enclosure along with a solid state preamplifier (Therapeutics Unlimited model 544, Iowa City, IA – no longer in business). Motion data were initially taken with an electronic goniometer (SG150B, Biometrics Ltd, Newport, UK) that was synchronized with the EMG system. After the first 30 participants, both measurement systems were replaced with a wireless EMG system with integrated accelerometers to provide motion data (Delsys Inc. Trigno Wireless System, Natick, MA). The major difference between the two EMG systems is that the original system had fixed-distance electrodes, whereas the current Delsys system uses two separate electrodes. The EMG and motion data are recorded using the Motion Monitor workstation with a sampling rate of 1000 Hz.

**Figure 2:**
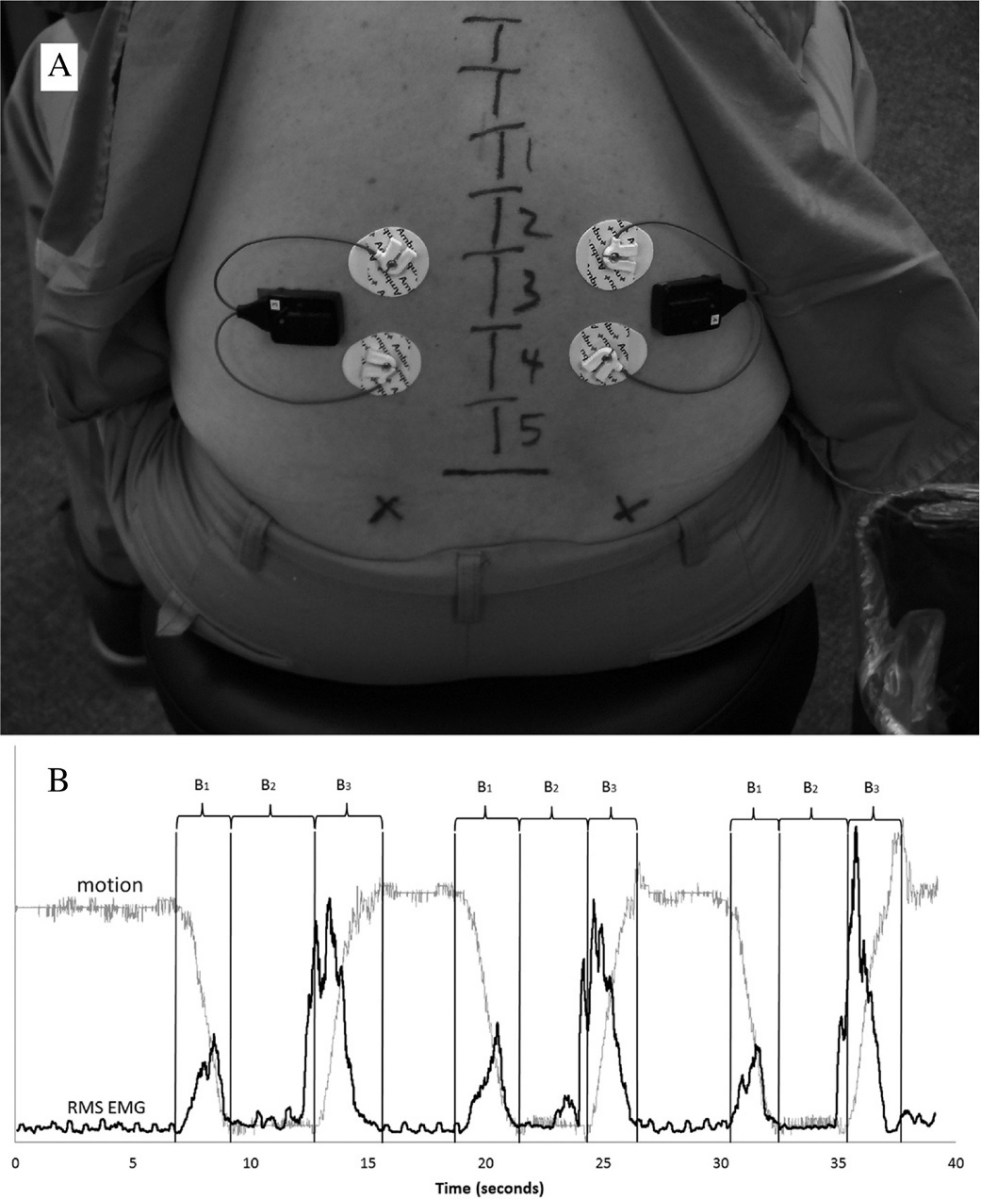
**Illustration of EMG electrode placement (A) and example EMG and motion signals (B).** Root-mean-square (RMS) EMG data and motion data are scaled according to the maximum of RMS EMG data **(B)**. B contains 3 cycles of flexion (B1), holding in position while fully flexed (B2), and extension (B3).

FRP measurements take place pre and post treatment during TV1, TV5, and TV12. The skin over the lumbar paraspinal muscles is prepared by vigorously rubbing the skin with an alcohol wipe. The participant, seated on a stool, leans forward while disposable, self-adhesive, pre-gelled surface EMG Ag/AgCl snap electrodes (Disposable ECG electrode white foam type, 36 mm in diameter, AMBU Inc., Copenhagen, Denmark) are placed on the skin over the mid-lumbar paraspinal muscles (Figure 2). Specifically the electrodes are placed with one above and one below the L3 level on each side with approximately 1 cm vertical distance between the edges of the electrodes in a semi-flexed position such that when the participant sits erect, the electrodes do not touch. Because the electrodes are removed to enable HVLA-SM, the outline of each electrode is drawn with a skin marking pen so new ones can be placed in the same location for the post treatment measurements.

Participants are instructed to place their feet shoulder width apart with their arms hanging loosely at their side. Keeping their knees straight, they bend forward as far as they can and hold that position for 3 seconds, then return to the upright standing position. Participants perform this movement once as practice and to allow research personnel to ensure proper functioning of the system. The participant then performs the procedure 3 times in a continuous manner while the EMG and motion data are collected.

*Data reduction*: In order to quantify the degree to which the FRP is present, two different forms of a flexion-relaxation ratio (FRR) are used [31, 32]. One is calculated by dividing the maximum root mean square (RMS) EMG activity level during flexion (while bending forward) by the lowest mean EMG activity as measured over a one second interval during the fully flexed phase. Another FRR is similarly calculated by dividing the maximum RMS EMG activity level during extension (while returning to standing erect) by the same minimum. The beginning and end of the fully flexed phase for each cycle are determined from the plot of the motion data. A macro written in Visual Basic for Applications, within Microsoft Excel (Microsoft Corp., Redmond, WA), is used to identify the specific regions of the data plots. These are regions of full flexion, the regions in which the trunk is flexing forward, and the regions in which the trunk is extending while returning to the upright position. The macro allows the user to adjust the limits of the various regions of the plot in order to exclude occasional spurious spikes in the RMS EMG data.

In all, 12 FRRs are calculated each time the participant performs this test. A set of 3 FRRs is based on the maximum RMS EMG activity during flexion – 1 FRR for each of the 3 cycles from the left paraspinal muscles. Another set of 3 is made from the right paraspinal muscles. Similarly, 2 more sets of 3 are made: one based on the maximum RMS EMG activity levels during extension from the left and the other similarly from the right paraspinal muscles. The FRRs for the 3 cycles of each situation are averaged which yields 4 mean FRRs each time a participant performs this activity: a mean FRR from flexion and one from extension for the left and right side. Once the regions have been identified the macro performs the necessary calculations.

### Secondary outcome measures

#### DC self-reported record of SM delivery over the course of treatment

**Figure 3:**
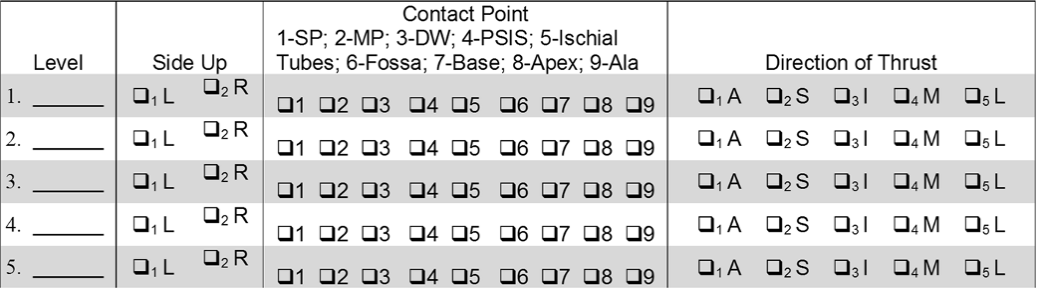
**Self-reported record for high velocity, low amplitude spinal manipulation.** Level: the segment over which the manual contact occurs (L1-5, sacrum, and sacroiliac joint); Side Up: side of body contacted by clinician providing trunk twist motion direction; Contact Point: manual contact of the thrusting hand; SP: spinous process; MP: mammillary process; DW: interspinous space 1 cm lateral to spinous processes; PSIS: posterior superior iliac spine; Ischial Tubes: Ischial tuberosity; Fossa: midpoint between ischial tuberosity and PSIS; Base: Medial aspect of the superior sacrum; Apex: inferior sacrum; Ala: sacral ala or lateral superior sacrum; Direction of thrust: direction of thrust applied by the thrusting hand; A: anterior; S: superior; I: inferior; M: medial; L: lateral.

DCs report all HVLA-SM delivered using a record log as illustrated in Figure 3. This information will allow us to better identify SM delivery patterns from the clinical perspective.

#### Contact force at the clinician’s thrusting hand

The measurement of contact force characteristics occurs as a part of the dynamic stiffness assessment (see more details below). The data reduction program used for segmental load is applied to extract variables from the contact force as measured under the clinician’s thrusting hand. These variables include the force exerted during the preload phase before the HVLA thrust, the loading rate, and peak force.

#### Segmental load

**Figure 4:**
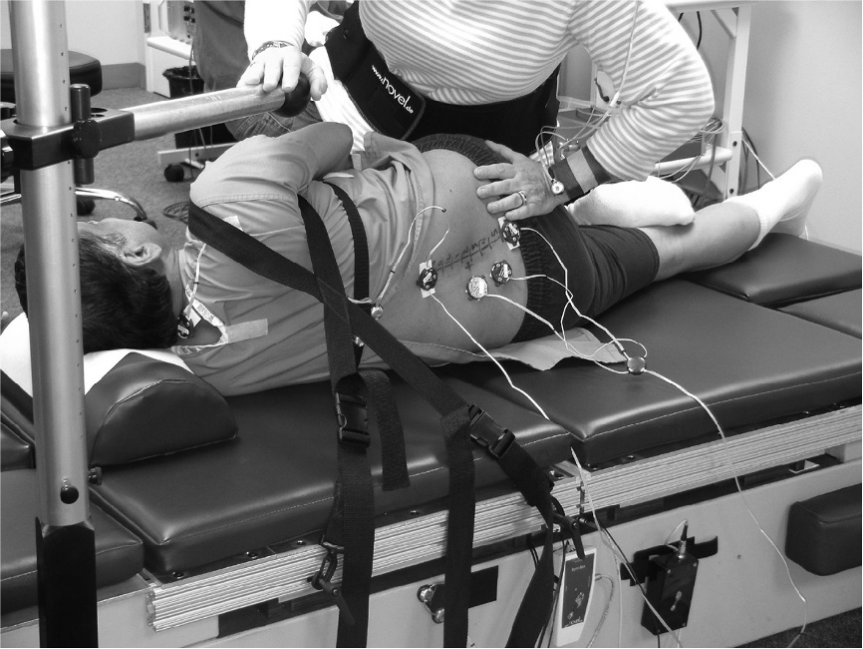
**Illustration of segmental load assessment during side-lying high-velocity spinal manipulation.**

The method for estimating lumbar spine segmental load proposed by Triano and Schultz [50] and Triano et al. [51] was adapted for use in the current study with two modifications. Rather than using a rigid chest panel to limit the participant’s upper body movement, we stabilize the upper shoulder with a shoulder strap crossed behind the participant (Figure 4). The strap restricts the predominant motion of the participants receiving HVLA-SM, which tends to include whole-body movement in the cephalic direction and a rolling movement of the lower trunk toward the clinician. The strap design attempts to mimic the clinician’s stabilization hand (non-thrusting hand). The strap is then secured to the force plate right under the participant’s thoracic cage such that force and moment occurring during the HVLA-SM procedure are monitored. The clinician’s non-thrusting hand is placed on a moveable rest located near but not touching the participants shoulder to approximate the typical position attained during the procedure and to help the clinician maintain balance (Figure 4). The location of the lumbar segment targeted by the contact hand is traced with three Optotrak smart marker rigid bodies during the HVLA-SM procedure instead of the one-time digitization during the preload phase used by Triano et al. [51]. As a result, it is possible to compensate for the effect that lumbar spine movement may have on segmental load.

The method described in Triano and Schultz [50] and Triano et al. [51], however, does not consider the effect of lumbar segment orientation on segmental load. As a result, only the magnitude of the segmental force and moment will be reported. To estimate three-dimensional segmental load, the location of the joint center and its orientation of the target lumbar segment need to be determined. We adopted the method developed by Splittstoesser [61] to fulfill this task. The procedure involves the creation of individualized lumbar spine models in the standing posture using skin marks over key spinal landmarks [62]. Additionally four Optotrak smart marker rigid bodies are placed at C7, T7, T10, and S1 to estimate the orientation of the target segment using the individualized lumbar spine model (Figure 4) [61]. The limitation of Splittstoesser’s method is that it is only designed to estimate sagittal plane motion. It does not account for lumbar spine axial rotation or lateral flexion, which may also occur during HVLA-SM. Thus, investigation of the effects of segment orientation on segmental load estimation in this study is exploratory.

*Data reduction*: A custom-written MATLAB program is applied for segmental load data reduction. Variables extracted from the segmental load data include the force and moment during the preload phase of the HVLA-SM, loading rate, and peak force and moment.

#### Dynamic stiffness

To investigate dynamic spinal stiffness (i.e. the displacement, acceleration and force characteristics in time), a mixed approach is applied to measure: the movement of the doctor’s thrusting hand using a triaxial accelerometer (Model 356A17, PCB Piezotronics Inc., Depew, NY) with a sampling rate at 1000 Hz; the Optotrak motion capture system with a sampling rate at 100 Hz; and the arithmetic summation of the thrusting force over the area at the base of the palm using a thin pressure pad (Pliance system, Novel Electronics, St Paul, MN) with a sampling rate of 50 Hz. Our previous study demonstrated that a sampling rate at 50 Hz is sufficient for acquiring contact force during HVLA-SM [36]. Two characteristics of dynamic spinal stiffness are computed using the force/displacement/acceleration-time profiles: 1) apparent mass (M) that takes into account the force applied (F) and the resulting acceleration (a) of the contact interface (M = F/a); and 2) driving-point mechanical impedance (Z) that takes into account the force applied (F) and the resulting velocity (v) of the contact interface (Z = F/v). The dynamic spinal stiffness measures are obtained during TV3 and TV9.

### Data collection, management and quality control

A customized submission, tracking and reporting web-based system was developed for the study. It is comprised of multiple sub-modules integrated into one comprehensive study management web application that includes sub-modules for computer assisted telephone interviewing, participant eligibility checking, participant tracking and report generation. The ASP. NET web application elements were programmed in C# and Structured Query Language (SQL) using Microsoft Visual Studio 2010 (Microsoft, Redmond, WA, USA) and Microsoft SQL Server Management Studio. User-friendly data entry interfaces were programmed with appropriate participant flow restrictions, validation schemes and skip patterns. The system uses a Project/Users Permissions System to control project personnel access to web modules. The web system is password-protected and uses a Microsoft SQL Server database platform to store all data.

Information is collected at every stage of recruitment, and throughout treatment and assessment so that the patient flow can be reported according to the Consolidated Standards of Reporting Trials guidelines [63]. We collect recruitment source, total number of responses per recruitment source, the resolution of these responses (ineligible, refused or enrolled), the number of withdrawals and reason for withdrawal, and the number of participants completing the study. For each enrolled participant, we track compliance to the treatment protocol and the assessment data that were collected at each visit.

Participant self-report questionnaires and clinician-reported SM delivery records are paper-based forms with unique participant identification numbers. Study coordinators have oversight for all paper data collection forms, log each completed form into a form tracking interface of the web system and submit data forms for key-entry weekly. These forms are double key-entered by trained data entry clerks in an MS Windows program using range and validation checks to improve accuracy and are stored in locked filing cabinets. The electronic biomechanical data are stored on a password protected network file server. Data reduction is completed within 2 weeks of data collection, transferred to the data manager in Microsoft Excel workbooks, and then uploaded to Microsoft SQL Server and stored with all other project-related data.

Data management and quality control of all data are performed using SQL views, stored procedures and real-time, web-based reports. Automated reports are viewed by the data manager to determine if quality improvement actions must occur, such as improved documentation, protocol revisions or personnel retraining. Final project datasets of combined web, paper and biomechanical data are assembled by transferring data from Microsoft SQL Server to SAS System for Windows (Release 9.3, SAS Institute Inc., Cary, NC, USA). Back-up tapes of the network drive are produced nightly.

### Protection of human subjects and assessment of safety

#### Protection of human subjects

The study protocol was approved by the Palmer College of Chiropractic IRB.

#### Data and Safety Monitoring Committee (DSMC)

This trial is being monitored by an independent DSMC comprised of epidemiologists with expertise in LBP and CAM clinical trials, a biostatistician and a doctor of chiropractic. The DSMC’s role is to provide scientific and ethical oversight by evaluating the following data provided to them on a semi-annual basis: participant recruitment, accrual, and retention; adverse events (AEs); protocol deviations; data monitoring; and participant characteristics. To further monitor participant safety, serious AEs are reported to the chair of the DSMC within 5 business days. The DSMC makes recommendations to the Principal Investigator and the Office of Clinical and Regulatory Affairs at the National Center for Complementary and Alternative Medicine regarding continuation, termination, or other modifications to the study.

#### Adverse events

An IRB-approved AE grading and reporting protocol defines the process by which AEs are monitored and categorized for this study. This protocol also outlines when and how participant safety concerns are reported to the IRB, DSMC and funding agency. An AE is any untoward medical occurrence that may present itself during the conduct of the study and which may or may not have a causal relationship with the study procedures [64]. A serious AE is defined as any adverse event resulting in any of the following outcomes: death, a life-threatening adverse experience, hospitalization or prolongation of existing hospitalization, a persistent or significant disability/incapacity, or a congenital anomaly/birth defect resulting from a pregnancy. Participants are asked about adverse experiences at each TV and instructed to contact study personnel if they experience significant pain, discomfort or distress that they believe may be associated with treatment.

### Statistical analysis

SAS System for Windows and R (http://www.r-project.org/) will be used for data analysis. Descriptive statistics of participant baseline characteristics and all measurements for each of the 3 assessment times will be calculated. The 3 different methods of collecting spinal stiffness will be compared with intraclass correlation coefficients. The general approach to data analysis will be to first summarize the variations in the physiological and kinetic measures using hierarchical linear regression models and then to use that information to determine the most appropriate statistical methods, including linear mixed regression models [65] and conditional linear mixed regression models [65, 66].

For Specific Aim 1, the variations in physiological measures will be summarized using a hierarchical linear regression model with a random effect for participant. If there is a non-significant random effect for participant, then this estimated random effect will be considered a sufficient summary of the physiological measures over time and linear mixed regression models will be used to examine the association between that random effect and the patient-centered outcome variables. If there is a significant random effect for participant, then there is variation in the measures over time. In this case, a linear mixed regression model with time-varying covariates will be fit to assess the association between the physiological measurements at a particular time point and patient-centered outcomes at the subsequent visit(s).

For Specific Aim 2, the variations in kinetic measures will be summarized using a hierarchical linear regression model with random effects for clinician and for participant nested within clinician. Intraclass correlation coefficients will be computed to establish if there is substantial consistency within clinician within participants and within clinician across participants and if there is sufficient variation between clinicians to allow evaluation of how variations in kinetic measures can predict patient-centered outcomes. If the random effect for participant within clinician is non-significant, or the intraclass correlation coefficient is 0.7 or higher, then the estimated random effect for clinician will be considered a sufficient summary of the delivery of care by an individual clinician. In this case, the association between this random effect and patient-centered outcomes will be evaluated using a linear mixed regression model. If there is not sufficient consistency within clinician between patients, but there is consistency within a clinician within participants, it will indicate that clinicians vary their care by participant but provide a consistent level of care across the course of treatment. In this case, the linear mixed regression model will be used to estimate the association between random effects specific to the clinician/participant combination and patient-centered outcomes. If consistency within clinicians and within participants cannot be established, this indicates substantial variation in the care provided across time by clinicians within a participant. In this case, we will fit a linear mixed regression model with time-varying covariates to assess the association between the kinetic measurements at a particular time point and patient-centered outcomes at the subsequent visit(s).

In the analysis for both specific aims 1 and 2, the complexity introduced by modeling with time-varying covariates may be better served with conditional linear mixed models. Regardless of what model is chosen, alternative models will be fit to assess the sensitivity of the statistical model to any assumptions about consistency.

#### Sample size

A minimum of 80 participants will be enrolled in the current study. The sample size should be sufficient to obtain preliminary estimates of variability and effect size through our planned data analysis, taking into consideration the possibility of dropouts and technical issues that may occur given the rigor and complexity of this study protocol.

## Discussion

**Figure 5:**
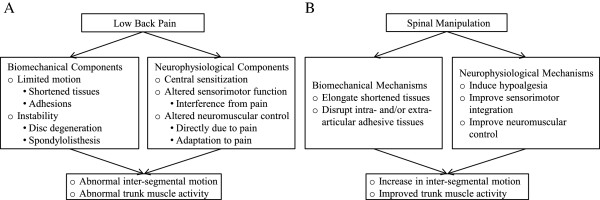
**Putative effects of low back pain (A) and spinal manipulation (B).**

The primary objective of the current study is to examine the predictive value of spinal stiffness and FRP for patient-centered outcomes in those with chronic LBP and treated with HVLA-SM. However, it also allows us to examine two domains that may contribute to the therapeutic effects of SM (i.e. neurophysiological and biomechanical processes). Analyzing both spinal stiffness and FRP measures in LBP patients undergoing SM treatment may help elucidate the contributions of passive (skeletal, ligamentous and disc structures) and active (muscles) structures during and following HVLA-SM, thus providing evidence for suspected therapeutic mechanisms [67].We hypothesize that changes in lumbar spine stiffness may be in part attributed to material changes in spinal tissues and altered paraspinal muscle activity induced by SM. Figure 5 illustrates two potential mechanistic pathways (biomechanical and neurophysiological) that are shared as putative mechanisms of LBP, whereby SM may lead to altered spinal stiffness and flexion-relaxation phenomenon in LBP patients. Knowledge gained from this study will further our understanding of the neuro-biomechanical mechanisms influenced by SM and may provide objective assessment measures for use in future clinical trials.

## Abbreviations

AE: Adverse events
BL1: Baseline Visit 1
BL2: Baseline Visit 2
C1-C7: T1-T12, L1-L5, and S1-S3: Segments of cervical, thoracic, and lumbar spines, and sacrum
DC: Doctors of chiropractic
DSMC: Data and safety monitoring committee
EMG: Electromyography
FRP: Flexion-relaxation phenomenon
FRR: Flexion-relaxation ratio
GSV: Global stiffness variation
HVLA-SM: High velocity low amplitude spinal manipulation
IRB: Institution Review Board
LBP: Low back pain
NIH: National Institute of Health
NRS: Numerical rating scale
PROMIS: Patient Reported Outcomes Measurement Information System
QTF: Quebec Task Force (classification for Spinal Disorders)
RMDQ: Roland Morris Disability Questionnaire
SM: Spinal manipulation
RMS: Root-mean-square
SQL: Structured Query Language
TV: Treatment visit
VAS: Visual analog scale.
